# Preoperative Diagnostic Angiogram and Endovascular Aortic Stent Placement for Appleby Resection Candidates: A Novel Surgical Technique in the Management of Locally Advanced Pancreatic Cancer

**DOI:** 10.1155/2015/523273

**Published:** 2015-09-28

**Authors:** N. Trabulsi, J. S. Pelletier, C. Abraham, T. Vanounou

**Affiliations:** ^1^Division of General Surgery, Jewish General Hospital, McGill University, 3755 Côte-Ste-Catherine Road, Montreal, QC, Canada H3T 1E2; ^2^Department of Surgery, King Abdulaziz University, P.O. Box 80215, Jeddah 21589, Saudi Arabia; ^3^Division of Vascular Surgery, Jewish General Hospital, McGill University, 3755 Côte-Ste-Catherine Road, Montreal, QC, Canada H3T 1E2

## Abstract

*Background*. Pancreatic adenocarcinoma of the body and tail usually presents late and is typically unresectable. The modified Appleby procedure allows resection of pancreatic body carcinoma with celiac axis (CA) invasion. Given that the feasibility of this technique is based on the presence of collateral circulation, it is crucial to confirm the presence of an anatomical and functional collateral system.* Methods*. We here describe a novel technique used in two patients who were candidates for Appleby resection. We present their clinical scenario, imaging, operative findings, and postoperative course.* Results*. Both patients had a preoperative angiogram for assessment of anatomical circulation and placement of an endovascular stent to cover the CA. We hypothesize that this new technique allows enhancement of collateral circulation and helps minimize intraoperative blood loss when transecting the CA at its takeoff. Moreover, extra length on the CA margin may be gained, as the artery can be transected at its origin without the need for vascular clamp placement.* Conclusion*. We propose this novel technique in the preoperative management of patients who are undergoing a modified Appleby procedure. While further experience with this technique is required, we believe that it confers significant advantages to the current standard of care.

## 1. Introduction

According to the most recent report from the SEER database, an estimated 46,420 new cases of pancreatic cancer will be diagnosed in the United States in 2014, with 39,590 related deaths. Despite recent advances in neoadjuvant and adjuvant treatment modalities, the prognosis is still dismal, with an estimated 5-year survival rate of 6.7% [1]. Unlike tumors that arise in the head of the pancreas, which present with obstructive jaundice, those that arise in the body and tail usually present late and are typically unresectable [2–4].

The NCCN Guidelines still consider tumors with celiac axis invasion to be unresectable; nonetheless, there has been a relatively recent trend towards a more aggressive surgical approach in the management of locally advanced pancreatic cancer [5–8].

In the 1950s, Lyon Appleby first described the feasibility of celiac axis resection for locally advanced gastric cancer, which relied on the presence of an effective superior mesenteric artery (SMA) collateral circulation to the common hepatic artery (CHA) through the gastroduodenal artery (GDA) or on the presence of aberrant anatomy [9]. In 1976, Nimura et al. adopted Appleby's understanding of the utility of the collateral SMA circulation and applied the technique to the surgical treatment of locally advanced pancreatic body carcinoma with celiac axis or branch invasion [10]. This technique was further modified in the 1990s to preserve the stomach via sparing of the GDA and the right gastroepiploic artery, known as the “modified Appleby resection” [11, 12].

Given that the feasibility of this advanced surgical technique is entirely based on the presence of collateral circulation or aberrant anatomy, it is crucial to objectively confirm the presence of an anatomical and functional collateral system. Many different methods have been described to assess the presence of collateral flow. The original description by Appleby included an intraoperative assessment of collateral flow through the GDA after manually occluding (clamping) the CHA proximal to the takeoff of the GDA. This technique has been adopted by many surgeons and is by far the most commonly used method. Others have described assessment by intraoperative Doppler ultrasonography to complement manual digital assessment as a more objective method [13]. The drawback of both techniques is the necessity of performing an exploratory laparotomy in order to do the assessment.

Preoperative assessment of vascular anatomy and collateral circulation could provide crucial information to help with decision making. Computed tomography (CT) with angiography is a sensitive method of capturing a “snapshot” of the arterial anatomical system to illustrate the patient's vascular anatomy, including aberrant variants [14]. An angiogram, on the other hand, not only provides more of a “real-time” demonstration of the patient's vascular anatomy, but is also the only reliable way to conclusively demonstrate, preoperatively, the presence or absence of collateral flow in the case of celiac trunk occlusion. The use of an angiogram to reliably assess sufficient visceral flow through collateral vessels has been tested and described during the endovascular repair of thoracoabdominal aneurysms, where occasionally coverage of the celiac artery (CA) is necessary and has been shown to be feasible and safe [15, 16].

We here describe a novel technique used in two cases of pancreatic cancer at our institution that were candidates for Appleby resection. Both patients had a preoperative angiogram for assessment of anatomical circulation and placement of an endovascular stent to cover the CA once confirmation of adequate collateral flow was obtained. This procedure not only helps enhance collateral circulation but, theoretically, it may help to minimize intraoperative blood loss when transecting the CA at its takeoff. Moreover, extra length on the CA margin may be gained as the artery can be transected at its origin without the need for vascular clamp placement.

## 2. Reports

### 2.1. Case Number 1

**Figure 1:**
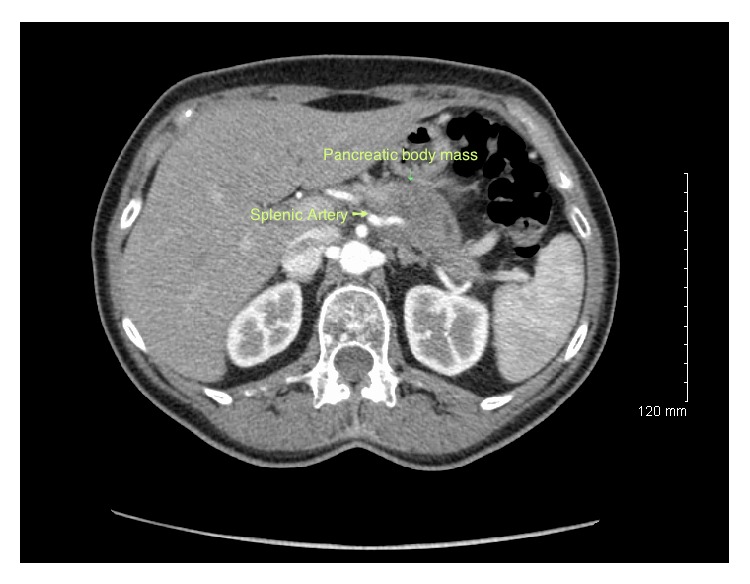
Computerized tomography scan of the abdomen and pelvis of the first patient demonstrating a pancreatic body and tail mass with circumferential splenic artery encasement.

The first case is a 63-year-old woman with diabetes mellitus and no surgical history, who was found to have pancreatic adenocarcinoma during an investigation of her epigastric pain. Her preoperative CT scan showed a pancreatic body and tail mass, measuring 3.4 × 3.2 × 3.5 cm (Figure 1). The primary mass encircled the splenic artery circumferentially, focally abutting the stomach, as well as the anterosuperior aspect of the left renal vein. The splenic vein was occluded. There was no encasement of the SMA or the portal vein. An ultrasound-guided biopsy of the mass confirmed an adenocarcinoma compatible with a pancreatic origin. Her CT scan of the chest demonstrated no evidence of metastatic disease.

**Figure 2:**
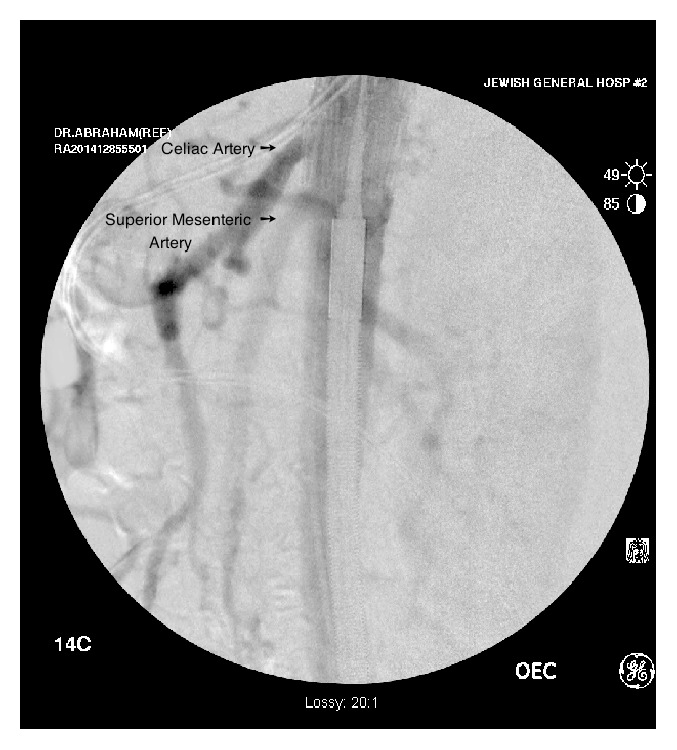
Aortic angiogram of the first patient demonstrating endovascular stent placement with coverage of the celiac artery.

The patient underwent neoadjuvant treatment (FOLFIRINOX followed by radiation therapy and Gemcitabine). A follow-up CT scan of the chest, abdomen, and pelvis after her neoadjuvant treatment showed stable disease. Three weeks later, in November 2014, she underwent an angiogram with an endovascular stent placement. Filling of the small bowel mesentery after the CA angiogram confirmed the presence of collateral circulation to the SMA. An endovascular stent (24 mm/42 mm proximal extension graft [Renu Stent Graft, Cook Medical]) was placed to cover the CA, as illustrated in Figure 2.

Three weeks later, the patient underwent a subtotal pancreatectomy with en bloc celiac axis resection (modified Appleby resection) with splenectomy, en bloc left adrenalectomy, and partial gastrectomy. During the exploration, it was noted that good collateral system development was evident from the size of the gastroepiploic vessels. Demonstration of continued flow to the proper hepatic artery after clamping the CHA proximal to the GDA takeoff was accomplished by using intraoperative Doppler ultrasonography. We ligated the left gastric artery, splenic artery, and CHA. The CA was clamped and suture ligated with a 3.0 Prolene suture.

The patient's postoperative course was unremarkable and she was discharged home on postoperative day 4. Her final pathology report showed residual ductal adenocarcinoma moderately to poorly differentiated in the tumor bed measuring 3 cm with estimated viable tumor cell cellularity of 15%. All margins were negative and there was no evidence of lymph node involvement (0/14 lymph nodes).

### 2.2. Case Number 2

**Figure 3:**
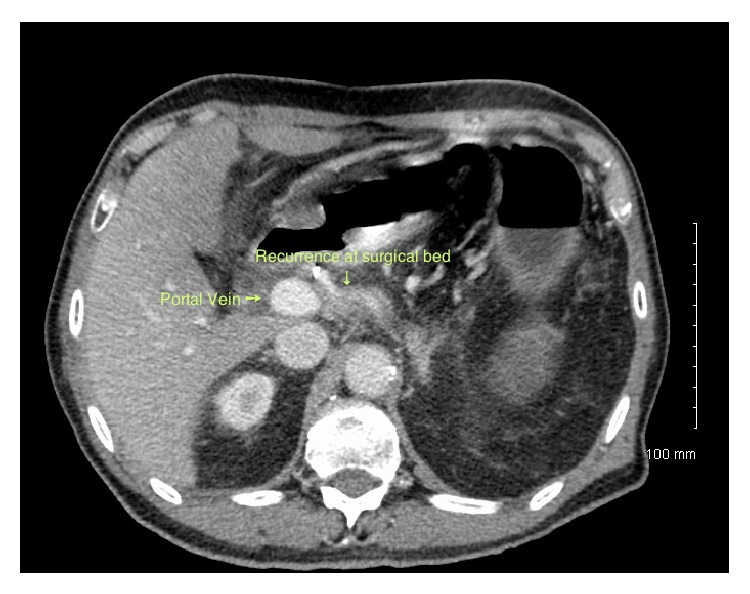
Computerized tomography scan of the second patient showing the mass at the surgical bed with encasement of the portal vein.

The second case is a 72-year-old man with recurrent pancreatic cancer. His original diagnosis of pancreatic cancer was in 2007 in an outside institution, after which he underwent a distal pancreatectomy. He was seen postoperatively at our institution for adjuvant treatment and surveillance, during which time he was found to have a local recurrence on imaging. His annual CT scan showed a soft tissue mass, located between the hepatic artery and the portal vein, which encased the former vessel. Its maximal diameter was measured at 2.5 × 2 cm, with an adjacent necrotic node measuring 1.2 × 1.5 cm (Figure 3). On positron emission tomography, only focal mild hypermetabolism was noted at the site of the suspected recurrence with no evidence of metastatic disease, which was confirmed on a CT chest.

The patient underwent neoadjuvant therapy (Gemcitabine with radiation therapy followed by Gemcitabine-Oxaliplatin). A CT scan to assess response showed stable disease.

**Figure 4:**
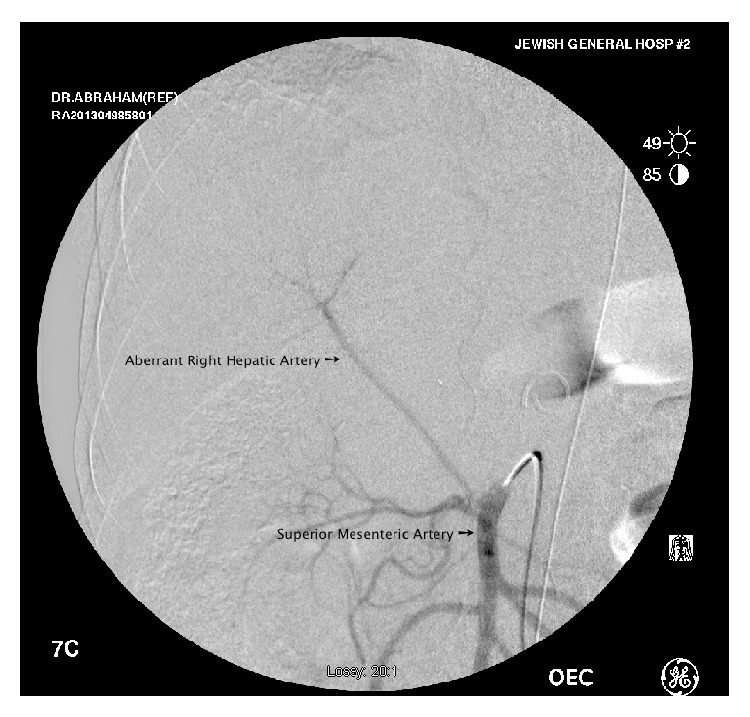
Angiogram of the second patient demonstrating an aberrant right hepatic artery originating from the superior mesenteric artery.

In April 2013, the patient underwent an angiogram that detected a replaced right hepatic artery originating from the SMA (Figure 4). An endovascular stent (32 mm proximal extension graft [Renu Stent Graft, Cook Medical]) was placed to cover the CA. Three weeks later, the patient underwent an exploratory laparotomy, which revealed the mass in the surgical bed, where the CHA was completely engrossed by the tumor with a margin of less than 1 cm before the takeoff of the GDA. Confirmation of effective collateral flow after clamping the CHA was demonstrated and further confirmed with intraoperative Doppler sonography. The mass was resected en bloc after clamping the CHA and transecting it just proximal to the GDA. In this patient's case, we were required to resect the CA flush with the aorta to ensure gross negative margins; this was made less complicated because of the presence of the endovascular stent. A partial portal vein resection was also performed with a longitudinal repair.

The patient did well postoperatively with no complications and was discharged home on postoperative day 5. His final pathology report showed moderately differentiated adenocarcinoma measuring 2.5 cm with focal involvement of resection margin. Twenty lymph nodes were examined and one was found to be involved by adenocarcinoma.

This patient was seen recently for his 18-month follow-up and was doing very well with no clinical or radiological evidence of recurrence of his disease.

## 3. Discussion

With this report, we introduce a novel technique in candidates for a modified Appleby resection. The use of this technique offers several advantages over standard techniques. The use of a preoperative angiogram offers real-time visualization of the patient's circulation with reliable assessment of collateral circulation. This can be demonstrated by viewing hepatic flow during a selective SMA angiogram or by viewing mesenteric flow during a selective CA angiogram. Another technique that could also be used (but was not used in our cases) is the balloon occlusion test, in which the CA can be temporarily occluded and then collateral flow demonstrated during a selective SMA angiogram [16]. In certain patients, such as in our second case, aberrant hepatic artery anatomy can be demonstrated during the angiogram. In the event of demonstration of inadequate collateral circulation, a more aggressive combined and technically demanding resection with hepatic artery reconstruction could be discussed with the patient [17–19].

Preoperative CA obstruction not only confirms the adequacy of the collateral circulation prior to performing a major resection but may also allow for the patient to improve his/her collateral flow preoperatively and potentially decrease the risk of postoperative ischemic complications, a well-recognized risk with this procedure [20]. Keskitalo and colleagues demonstrated in animal lab that experimental acute CA occlusion in dogs increased the collateral blood flow to 30% immediately and to 50% after three hours [21]. This occlusion-induced increase of pressure within the preexisting collateral system triggers arteriogenesis (defined as the growth of functional collateral arteries from preexisting arterioarteriolar anastomoses) and is mediated by the proliferation of vascular smooth muscle cells [22, 23]. This neocollateral network was found in animal labs to have partial flow detectable as early as few days with the bulk of network development occurring by 3 weeks [24, 25].

The presence of the stent also allows the resection of the CA as close as possible to its takeoff without the need to place a vascular clamp. This is a major advantage as compared to the technique of coiling the common hepatic artery preoperatively as described by Takasaka et al. [26]. Thus, this will improve the ability to safely obtain negative margins and limit intraoperative blood loss during this part of the procedure.

As with any innovated surgical technique, potential hazards should be weighed against benefits. It is true that an angiogram with endovascular stent placement is on its own an invasive procedure with potential complications; however, if done by experts in the field in properly selected patients, it can be deemed safe [27–29].

## 4. Conclusion

We therefore propose this novel technique in the preoperative management of patients undergoing a modified Appleby procedure. While further experience is required, we believe that it confers significant advantages to the current standard of care.
